# Automated UPDRS Gait Scoring Using Wearable Sensor Fusion and Deep Learning

**DOI:** 10.3390/bioengineering12070686

**Published:** 2025-06-24

**Authors:** Xiangzhi Liu, Xiangliang Zhang, Juan Li, Wenhao Pan, Yiping Sun, Shuanggen Lin, Tao Liu

**Affiliations:** 1 The State Key Laboratory of Fluid Power and Mechatronic Systems, School of Mechanical Engineering, Zhejiang University, Hangzhou 310027, China; liuxiangzhi@zju.edu.cn (X.L.); xlzh@zju.edu.cn (X.Z.); panwh@zju.edu.cn (W.P.); 3200102539@zju.edu.cn (S.L.); 2 Beijing Research Institute of Mechanical and Electrical Engineering, Beijing 102202, China; lijuan_208suo@126.com; 3 The Second School of Clinical Medicine, Zhejiang Chinese Medical University, Hangzhou 310053, China; sunyiping@zcmu.edu.cn

**Keywords:** automatic gait scoring, unified Parkinson’s disease rating scale, electromyography, inertial measurement unit, sensor fusion

## Abstract

The quantitative assessment of Parkinson’s disease (PD) is critical for guiding diagnosis, treatment, and rehabilitation. Conventional clinical evaluations—heavily dependent on manual rating scales such as the Unified Parkinson’s Disease Rating Scale (UPDRS)—are time-consuming and prone to inter-rater variability. In this study, we propose a fully automated UPDRS gait-scoring framework. Our method combines (a) surface electromyography (EMG) signals and (b) inertial measurement unit (IMU) data into a single deep learning model. Our end-to-end network comprises three specialized branches—a diagnosis head, an evaluation head, and a balance head—whose outputs are integrated via a customized fusion-detection module to emulate the multidimensional assessments performed by clinicians. We validated our system on 21 PD patients and healthy controls performing a simple walking task while wearing a four-channel EMG array on the lower limbs and 2 shank-mounted IMUs. It achieved a mean classification accuracy of 92.8% across UPDRS levels 0–2. This approach requires minimal subject effort and sensor setup, significantly cutting clinician workload associated with traditional UPDRS evaluations while improving objectivity. The results demonstrate the potential of wearable sensor-driven deep learning methods to deliver rapid, reliable PD gait assessment in both clinical and home settings.

## 1. Introduction

Parkinson’s disease is a prevalent movement disorder that imposes substantial burdens on both society and individual families. Accurate assessment of Parkinson’s disease is essential for formulating targeted treatment and rehabilitation strategies. By enabling early and precise intervention, such assessments can slow disease progression [1,2]. Gait analysis [3,4] is a critical component of Parkinson’s disease evaluation and has long attracted clinician attention. However, conventional clinical gait assessment remains reliant on rating scales such as the Unified Parkinson’s Disease Rating Scale (UPDRS). These scales require considerable clinician time and effort. Moreover, UPDRS gait scores for the same subject can vary by up to 25% when assessed by different clinicians [5], reducing inter-rater reliability.

With advances in sensor technology, instrumented gait analysis has emerged as an effective tool for automated Parkinson’s assessment. Although optical motion capture remains the gold standard for movement tracking, it has also been applied extensively in clinical gait studies. Wang et al. [6] employed a kernel extreme learning machine (KELM) to implement a fuzzy decision-making approach between successive frames of an optical motion capture system. They validated this on walking gait data from 76 participants and demonstrated its advantages for gait analysis. Anna et al. [7] further used an optical setup to classify the gait patterns of 15 healthy subjects and 15 Parkinson’s disease patients, thereby quantifying motor differences between the groups.

However, high cost and strict environmental constraints limit the clinical use of marker-based optical systems. As a low-cost alternative, many groups have adopted markerless, camera-based approaches. For example, Zachary et al. [8] compared a markerless system against a marker-based optical setup in 57 participants (including Parkinson’s patients), finding high consistency in spatial gait parameters. Rachneet et al. [9] used standard video recordings to classify gait in individuals with multiple sclerosis, Parkinson’s disease, and healthy elderly controls. They evaluated various machine learning classifiers and found that convolutional neural networks achieved the highest accuracy of 79.3%. Tan et al. [10] adopted a smartphone-based markerless method to extract features such as stride length, turning steps, turning duration, velocity, and cadence from healthy subjects and Parkinson’s patients. This approach achieved 89.39% accuracy. However, markerless methods remain sensitive to occlusions and limited camera field of view, which restricts long-term monitoring in clinical settings.

Wearable sensors—owing to minimal environmental constraints and low interference with natural movement [11,12]—are well suited for long-term gait monitoring. Dante et al. [13] extracted seven gait features from shank-mounted IMUs worn by over 100 participants and applied machine learning to screen for Parkinson’s disease, achieving 80% accuracy. Thomas et al. [14] demonstrated that a single wrist-worn IMU could detect freezing of gait in eleven Parkinson’s patients. These studies confirm the promise of IMU-based diagnostics but focus primarily on disease detection rather than UPDRS scoring.

In addition to IMUs, surface electromyography (sEMG) has seen growing application in Parkinsonian gait assessment. Recent advances in electrode materials and fabrication have greatly improved signal stability, signal-to-noise ratio, and wearer comfort. These innovations make sEMG a reliable, user-friendly modality for capturing subtle muscular activation patterns of early-stage Parkinson’s disease [15,16,17]. Christopher et al. [18] used a convolutional neural network on five-muscle sEMG recordings to classify Parkinson’s disease with 91.9% accuracy. Other work [19] achieved 99% diagnostic success using SVMs with radial basis function kernels, demonstrating that deep learning on EMG data can outperform traditional methods.

Most current wearable sensor research remains at the diagnostic level—distinguishing Parkinson’s patients from controls—while automated scoring of clinical scales is underexplored. Urs et al. [20] recorded eight-channel forearm EMG during a tapping task in 45 Parkinson’s patients and used random forest regression to predict UPDRS-III scores, achieving a correlation of 0.739. Rene et al. [21] mapped multiple UPDRS motor subitems using 2 dorsum-mounted IMUs per hand in 33 patients and 12 controls, attaining 94% accuracy. Han et al. [22] predicted UPDRS-III scores in 25 patients with 2 shank-mounted IMUs and a nonlinear mapping algorithm, achieving 84.9% accuracy. Nevertheless, these methods either involve complex sensor setups or lack sufficient precision for clinical adoption.

To address these challenges, we introduce a wearable-based UPDRS automatic scoring system. We designed a convolutional learning network to fuse signals from 2 lower limb IMUs and a four-channel sEMG array. We evaluated its performance on 10 healthy controls and 11 Parkinson’s patients. Our system achieved 92.8% accuracy in classifying UPDRS levels 0–2 while requiring only a simple walking task, thereby reducing assessment time and clinician effort compared to previous approaches.

## 2. Materials and Methods

### 2.1. Participant Selection and Protocols

This study was approved by the Medical Ethics Committee of the School of Biomedical Engineering and Instrument Sciences, Zhejiang University (Project ID: 2021-39). As shown in Table 1, 10 healthy volunteers and 11 Parkinson’s patients were recruited to participate in the gait experiments. All Parkinson’s patients underwent UPDRS assessment independently by three movement disorder specialists. We obtained the final score for each subject by averaging the three ratings to ensure evaluation accuracy. For safety and to minimize external interference, all participants were required to walk unaided (i.e., without canes or physical assistance), and thus, only UPDRS levels I and II were included; subjects with level III or higher—due to markedly impaired balance and inability to walk independently—were excluded from recruitment. Each participant performed three trials of a 10 m straight-line walk in a rehabilitation laboratory equipped with a floor-mounted measuring tape and minimal electromagnetic interference. To correct for any IMU mounting bias, subjects stood stationary for 5 s immediately before and after each walking trial.

**Figure 1:**
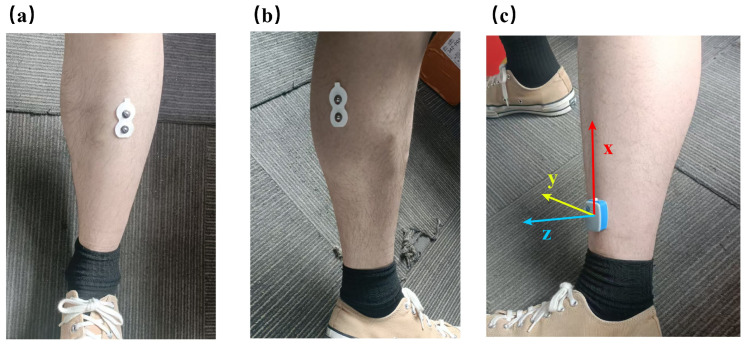
Sensor placement schematic. (**a**) Tibialis anterior EMG channel. (**b**) Gastrocnemius EMG channel. (**c**) IMU attachment on the lateral lower leg, 3–5 cm above the lateral malleolus.

The hardware setup consisted of four channels of 4 surface EMG and 2 IMU sensors, all acquired using Noraxon’s Ultium systems (Noraxon USA, Scottsdale, AZ, USA), with sensor positions depicted in Figure 1. The 2 IMUs were affixed to the lateral aspect of each lower leg, approximately 3–5 cm proximal to the ankle. This region exhibits relatively little underlying muscle mass, which facilitates firm sensor–skin coupling, minimizes motion artifacts arising from muscle sliding during gait, and has been shown to outperform trunk-mounted placements in both event detection accuracy and resistance to soft tissue vibration in Parkinson’s populations [23,24,25]. Four surface EMG channels recorded the activity of the tibialis anterior (TA) and gastrocnemius (GA) muscles of the lower leg—both of which are principal groups engaged during walking and established biomarkers of Parkinsonian gait. In particular, reduced TA activation during the stance and swing phases correlates strongly with UPDRS scores and responds to levodopa treatment [26], while GA burst timing at push-off provides complementary information on propulsion deficits [27].

### 2.2. Overall Framework of the Method

**Figure 2:**
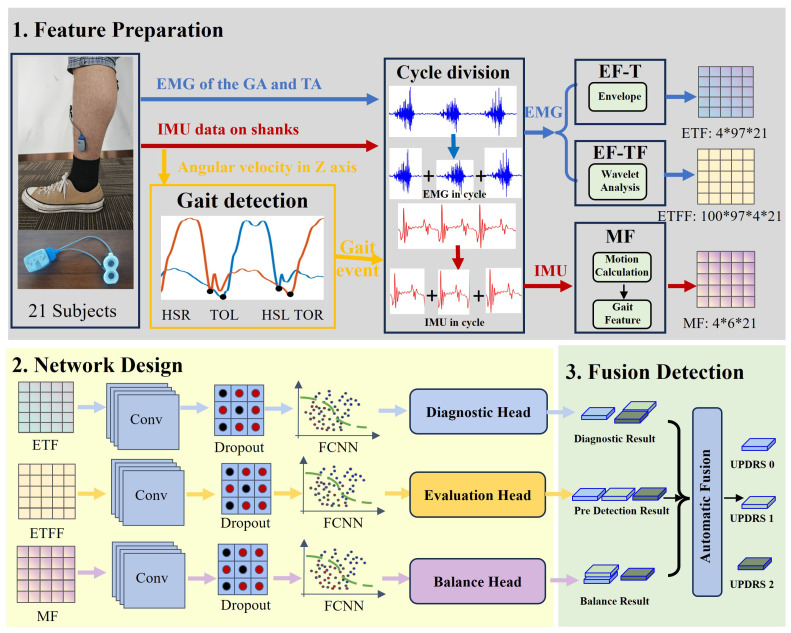
Overall diagram of the UPDRS automated evaluation method driven by multimodal data.

As illustrated in Figure 2, the overall framework of the proposed bimodal EMG–IMU-driven UPDRS automatic scoring method consists of three sequential stages. First, sagittal-plane angular velocity signals from an IMU mounted above the ankle are used to detect heel-strike and toe-off events, thereby segmenting the continuous time series data into individual gait cycles. EMG and IMU recordings are then processed separately as follows: for each gait cycle, the EMG signals are converted to their time-domain envelopes and analyzed via time–frequency wavelet transforms, and these cycle-wise representations are averaged to yield feature maps that reflect global lower limb muscle activation. Concurrently, key gait kinematics (e.g., stride length and swing ratios) are computed from the IMU and summarized—via a clustering algorithm—into features that characterize the overall gait pattern. Three parallel convolutional modules, which are widely used in other research [28], are constructed to process the feature map matrices generated in stage 1. Each module culminates in a distinct output “head”: a diagnostic head (predicting disease presence subitems), an evaluation head (estimating quantitative scores), and a balance head (assessing postural stability metrics). The outputs of the diagnostic, evaluation, and balance heads are then integrated using a fusion algorithm that emulates the multidimensional assessment performed by movement-disorder specialists. The fused result is mapped directly to an overall UPDRS-III score, yielding the system’s final automated evaluation of motor function.

### 2.3. Multi-Modal Features Extraction

#### 2.3.1. Motion Feature Extraction Based on IMU

Wearable sensor features for Parkinson’s disease UPDRS automatic scoring can be categorized into joint kinematic features (IMU-derived) and muscle time–frequency features (EMG-derived). To optimally capture Parkinsonian motor biomarkers across modalities, this study implements modality-specific feature extraction pipelines. Because IMUs directly measure acceleration and angular velocity at the attachment site, correct sensor placement is critical for data quality. However, in practice, mounting misalignments introduce inevitable discrepancies between the IMU coordinate frame and the lower limb motion. To mitigate this, we devised a simple static–dynamic calibration procedure.

In our static calibration step, we average the IMU’s acceleration readings during the first and last 5 s of quiet standing (no movement) to estimate the sensor’s orientation relative to gravity. Comparing this averaged vector to the expected gravity vector (i.e., [g, 0, 0] in the shank frame) allows us to compute misalignment angles about the y- and z-axes (Equation (1)).

(1) $$\left\{\begin{array}{c} a_{IMU}^{static}={\left[\begin{array}{ccc} {\bar{a}}_{x} & {\bar{a}}_{y} & {\bar{a}}_{z} \end{array}\right]}^{T}\\ a_{des}={\left[\begin{array}{ccc} g & 0 & 0 \end{array}\right]}^{T} \end{array}\right.$$

where $a_{IMU}^{static}$ denotes the mean of three axis accelerates in static period; ${\bar{a}}_{x}$, ${\bar{a}}_{y}$, ${\bar{a}}_{z}$ represent the mean accelerates in x-axis, y-axis, and z-axis, respectively; $a_{des}$ denotes the ideal accelerates of shank in static period, with acceleration only on the x-axis; and *g* is the acceleration due to gravity.

Through Equation (2), the mounting misalignment angles of the IMU about its local y- and z-axes can be obtained from the static IMU acceleration measurements and the target shank acceleration.

(2) $$\left\{\begin{array}{c} e_{pitch}=\tan^{-1}\left(\frac{-{\bar{a}}_{z}}{\sqrt{{{\bar{a}}_{x}}^{2}+{{\bar{a}}_{y}}^{2}}}\right)\\ e_{yaw}=\tan^{-1}\left(\frac{{\bar{a}}_{y}}{{\bar{a}}_{x}}\right) \end{array}\right.$$

where $e_{pitch}$ and $e_{yaw}$ denote the mounting error angles about the y- and z-axes, respectively.

However, static calibration based solely on the acceleration vector can only correct for misalignment about the y- and z-axes; it cannot correct for rotation about the IMU’s local x-axis (i.e., along the long axis of the shank), since such an offset does not alter the direction of the measured gravitational acceleration in static conditions. To address this, we have devised a dynamic calibration method as follows: by analyzing the displacement components along the IMU’s y- and z-axes in the transverse plane during gait, the misalignment about the x-axis can be derived. Because our experimental protocol requires all subjects to walk in a straight line, within a complete gait cycle (from one heel strike to the next), ideal displacement occurs only in the forward (y) direction. Therefore, the mounting error angle about the x-axis can be computed using Equation (3) as follows:

(3) $$e_{roll}=\tan^{-1}\left(\frac{x_{z}^{cycle}}{x_{y}^{cycle}}\right)$$

where $e_{roll}$ is the misalignment angle about the x-axis, and $x_{y}^{cycle}$ and $x_{z}^{cycle}$ are the displacements along the IMU’s y- and z-axes, respectively, over one full gait cycle after static calibration.

Through static and dynamic calibration (as shown in Algorithm 1), we corrected the IMU mounting errors, enabling high-precision acquisition of the raw sensor data and ensuring the accuracy of subsequent motion feature extraction. Regarding gait motion characteristics, we defined the following six core parameters to characterize this process, which are also commonly used in other studies [29,30]:
**Algorithm 1** Static–dynamic IMU calibration procedure**Input:** Sampling frequency $F_{s}=100\;$Hz, static duration $T_{s}=5\;$s, gait cycle *T*, raw IMU acceleration $a_{x},\;a_{y},\;a_{z}$, displacement $x_{z}^{cycle},\;x_{y}^{cycle}$ per gait cycle
**Output:** Mounting misalignment angles $e_{\mathrm{pitch}},\;e_{\mathrm{yaw}},\;e_{\mathrm{roll}}$
  1:Record acceleration $\left[a_{x}\left(t\right),\;a_{y}\left(t\right),\;a_{z}\left(t\right)\right]$ for $t\in [0,\;T_{s}]$ for static calibration and $t\in [t_{\mathrm{end}}-T,t_{\mathrm{end}}]$ for dynamic calibration  2:Compute means: $a_{IMU}^{static}={\left[\begin{array}{ccc} {\bar{a}}_{x} & {\bar{a}}_{y} & {\bar{a}}_{z} \end{array}\right]}^{T}$  3:Compute misalignment about y,z by static calibration method:$e_{pitch}=\tan^{-1}\left(\frac{-{\bar{a}}_{z}}{\sqrt{{{\bar{a}}_{x}}^{2}+{{\bar{a}}_{y}}^{2}}}\right)$$e_{yaw}=\tan^{-1}\left(\frac{{\bar{a}}_{y}}{{\bar{a}}_{x}}\right)$*static misalignment about y,z*  4:Collect displacement $x_{z}^{cycle},\;x_{y}^{cycle}$ over one gait cycle  5:Compute roll error by dynamic calibration method: $e_{roll}=\tan^{-1}\left(\frac{x_{z}^{cycle}}{x_{y}^{cycle}}\right)$*misalignment about x*  6:Apply rotations by to align IMU frame to anatomical frame**Output: **$e_{roll},e_{pitch},e_{yaw}$

(1) **Stride Length (SL)**: The anterior displacement of the ankle joint during one complete gait cycle.
(2) **Gait Cycle Duration (GC)**: The temporal duration of one complete gait cycle.
(3) **Swing Phase Ratio (SPR)**: The proportion of the gait cycle occupied by the swing phase.
(4) **Mean Gait Speed (MGS)**: The average walking speed over one gait cycle.
(5) **Maximum Foot Clearance (MFC)**: The maximum vertical (x-axis) displacement of the ankle joint—i.e., the peak foot lift—within one gait cycle.
(6) **Shank Range of Motion (SR)**: The angular excursion of the shank segment during one complete gait cycle.

#### 2.3.2. Time–Frequency Feature Extraction Based on EMG

We processed four-channel lower limb EMG signals by applying a 20–450 Hz bandpass filter and normalizing each channel. We then extracted the envelope—computing the analytic signal via the Hilbert transform, taking its magnitude, and smoothing—to capture temporal modulation (Equation (4)). Finally, wavelet analysis yielded a scalogram as the time–frequency feature matrix. Since band-pass filtering and normalization are widely used, well-established steps in EMG preprocessing, we have not expanded on their elementary formulations here to conserve space.

(4) $$env_{i}\left(t\right)=\sqrt{\varepsilon_{i}{\left(t\right)}^{2}+H{\left(\varepsilon_{i}\left(t\right)\right)}^{2}}$$

where $env_{i}\left(t\right)$ is the envelope amplitude of the *i*th channel at time *t*, $\varepsilon_{i}\left(t\right)$ is the filtered and normalized EMG amplitude of the *i*th channel at time *t*, and *H* denotes the Hilbert transform, which suppresses negative-frequency components to extract the instantaneous phase and amplitude information.

However, envelope extraction merely captures the temporal amplitude modulation of the raw EMG signal after the removal of high-frequency oscillations and does not preserve its spectral characteristics. Common frequency-domain methods such as the Fourier transform cannot accommodate phenomena with strong time-domain features—such as gait. Therefore, we employ wavelet analysis to achieve true time–frequency characterization of the EMG signals [31,32]. Specifically, we use cwt function with the Morlet wavelet in MATLAB R2024a as the mother wavelet; the resulting scalogram (i.e., the map of wavelet coefficients) then serves as the comprehensive time–frequency feature matrix of the EMG data.

### 2.4. Convolutional Network Design and Fusion Detection Method

#### 2.4.1. Multi-Module Convolutional Network Design

In the network design module, we implemented three independent convolutional subnetworks, each tailored to one of the three feature map modalities—IMU-derived gait kinematic maps, EMG time-domain envelope maps, and EMG time–frequency wavelet coefficient maps—for the tasks of gait diagnosis, comprehensive motor assessment, and balance impairment detection, respectively.

##### (1) Diagnosis Head

**Figure 3:**
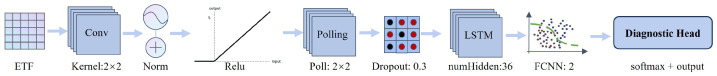
Convolutional network design of diagnostic head (EMG envelope → LSTM → classification).

Diagnosis head determines whether a subject is affected by Parkinson’s disease. Its input is a four-channel EMG envelope feature tensor (FTF) of size 4 × 97, in which each channel corresponds to the normalized time-domain envelope of one muscle over a single gait cycle. Because the envelope directly reflects muscular activation dynamics and has been shown to discriminate Parkinson’s patients from controls [33], the network (see Figure 3) comprises successive convolution batch normalization-ReLU blocks, followed by max-pooling and a dropout layer (*p* = 0.3) to mitigate overfitting. To capture temporal dependencies across the gait cycle, an LSTM layer is appended after the convolutional modules, enabling the model to learn sustained activation patterns that are characteristic of Parkinsonian muscle control.

##### (2) Evaluation Head

**Figure 4:**
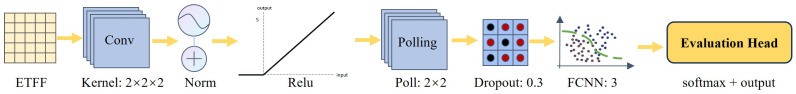
Convolutional network design of evaluation head (EMG wavelet coefficient map → convolutional network → classification).

Evaluation head predicts each subject’s UPDRS gait subscore. Its input is a four-channel EMG wavelet coefficient map (ETFF) of size 100 × 97 × 4, constructed by stacking the per-cycle time–frequency wavelet coefficients of each muscle. Owing to the richer spectral information and the three-dimensional nature of the ETFF, a 3-D convolutional network (see Figure 4) is employed to learn spatiotemporal correlations across time and frequency axes, culminating in a fully connected regression head that outputs the UPDRS-III gait score.

##### (3) Balance Head

**Figure 5:**
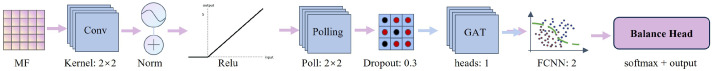
Convolutional network design of balance head (IMU gait feature → GAT → classification).

Balance head assesses whether a subject’s postural balance is significantly impaired. Its input is a 4 × 6 matrix of lower limb kinematic features (MF) derived from 2 ankle-mounted IMUs. Although such gait kinematics directly reflect balance deficits, early-stage Parkinson’s patients often exhibit only subtle alterations [34]. Therefore, in addition to convolutional feature extractors (see Figure 5), a self-attention mechanism is integrated to weight the six kinematic features according to their relevance to postural stability, thereby enhancing sensitivity to incipient balance impairments.

We trained and evaluated all three subnetworks using subject-wise cross-validation as follows: from a cohort of 10 healthy controls, 5 UPDRS-I patients, and 6 UPDRS-II patients, each iteration randomly designated 2 controls, 1 UPDRS-I, and 1 UPDRS-II subject as the test set, with the remaining subjects forming the training set. This procedure was repeated 21 times, ensuring that each subject appeared at least twice in the test set, and the aggregated performance over 84 test trials was taken as the final evaluation metric.

#### 2.4.2. Fusion Detection Method

In this study, we devised three subnetworks to estimate the final UPDRS-III rating, with the aim of leveraging multimodal information and emulating the multidimensional decision criteria used by clinicians. By integrating the outputs of these subnetworks, we establish mapping relationships that reflect real-world clinical reasoning, thereby improving the accuracy of the final UPDRS-III prediction.

Specifically, the *diagnosis head* functions as a virtual pre-assessment, analogous to an initial clinical screening. Its output is used to adjust the decision thresholds of the *assessment head* as follows: if a subject is classified as “Parkinson’s positive” by the diagnosis head, the threshold for Level 0 in the assessment head is raised, while those for Levels 1 and 2 are lowered, biasing the main assessment toward higher severity. Conversely, the *balance head* serves as a post-assessment check, quantifying balance impairment. If significant balance damage is detected, the threshold for Level 0 in the assessment head is greatly increased and that for Level 2 is greatly decreased, thus steering the final decision toward Level 2 when balance impairment is pronounced. All initial thresholds are set at 0.5 (i.e., an output probability greater than 0.5 indicates a positive decision for that level). The overall fusion mechanism is summarized as follows:

1. If the diagnosis head classifies a subject as healthy, the final assessment head cannot output Level 2; additionally, the Level 1 threshold is raised from 0.5 to 0.7.
2. If the diagnosis head classifies a subject as Parkinson’s positive, the Level 0 threshold in the assessment head is raised from 0.5 to 0.7.
3. If the balance head indicates no severe balance impairment, the Level 2 threshold in the assessment head is raised from 0.5 to 0.7.
4. If the balance head indicates severe balance impairment, the final assessment head cannot output Level 0; therefore, the Level 1 threshold is raised from 0.5 to 0.7.
5. The final rating must be exactly one of {0, 1, 2}; simultaneous assignment to multiple levels is not permitted.

This clinician-inspired fusion strategy ensures that the final UPDRS-III score reflects both broad diagnostic likelihood and specific functional impairments.

## 3. Results

Due to the limited number of participants in each subgroup (HC: n = 10; UPDRS-1: n = 5; UPDRS-2: n = 6), we perform a detailed effect size and post hoc power analysis on six key gait features to ensure that our findings remain robust despite small sample sizes. The Cohen’s d and post hoc power are computed for three pairwise comparisons (HC vs. PD1, HC vs. PD2, and PD1 vs. PD2) in Table 2. Notably, SR and SL/H yield very large effect sizes (d ≥ 1.6) with power ≥ 0.99 in both HC vs. PD2 and HC vs. PD1 comparisons, and SPR also exhibits d > 1.4 with power > 0.99 when distinguishing PD severity (HC vs. PD2 and PD1 vs. PD2). Although other features (e.g., GC, MGS) show smaller d values and lower power, the exceptionally high effect sizes and power for SR, SL/H, and SPR confirm that, even with only six UPDRS-2 subjects, these metrics can reliably detect group differences.

**Figure 6:**
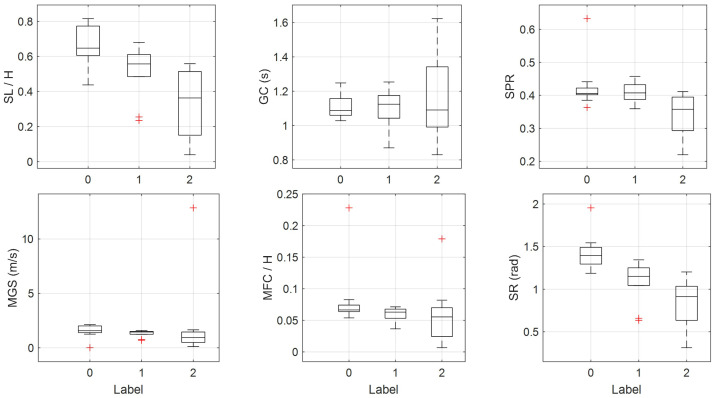
Box plots of six gait parameters at different UPDRS level. Red symbols denote outliers outside the whiskers that are not included in the boxplot statistics.

In Figure 6, we present box plots of six gait-kinematic features for all participants, stratified by UPDRS-III gait scores (0, 1, and 2). To remove the confounding effect of body stature, we normalize stride length (SL) and maximum foot clearance (MFC) by each subject’s height. The distributions for score 0 (healthy) and score 1 (Parkinson’s) show minimal separation, with only shank range of motion (SR) displaying a modest difference. However, their interquartile ranges still overlap substantially. By contrast, subjects with score 2 differ markedly from both score 0 and score 1 groups across all 6 features. This finding indicates that raw gait kinematics are more discriminative of severe balance impairment than of disease presence, thereby motivating our choice to use these features as inputs to the Balance-Impairment Detection Head.

**Figure 7:**
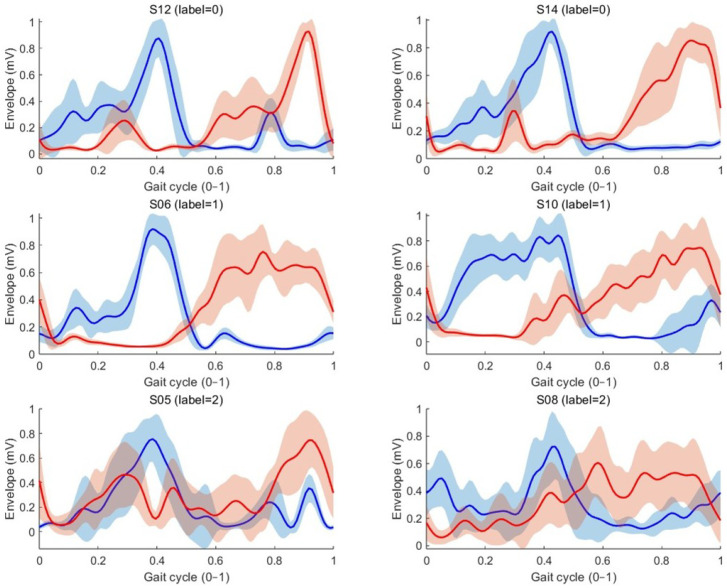
Normalized EMG envelopes of the left and right gastrocnemius muscles for for subjects 5, 6, 8, 10, 12, and 14. The blue and red line denotes the left and right gastrocnemius muscles, respectively.

Similarly, in Figure 7, we overlay the normalized EMG envelopes of the left and right gastrocnemius muscles for representative subjects: IDs 12 and 14 (score 0), 6 and 10 (score 1), and 5 and 8 (score 2). Each trace is time-warped to a single gait cycle, and the shaded regions denote cycle-to-cycle variability. Blue and red curves correspond to the left and right muscles, respectively. Healthy subjects (score 0) display substantially greater envelope symmetry and smoothness compared to both score 1 and score 2 groups. These observations support the use of EMG envelope features as primary inputs for the gait diagnosis head.

**Figure 8:**
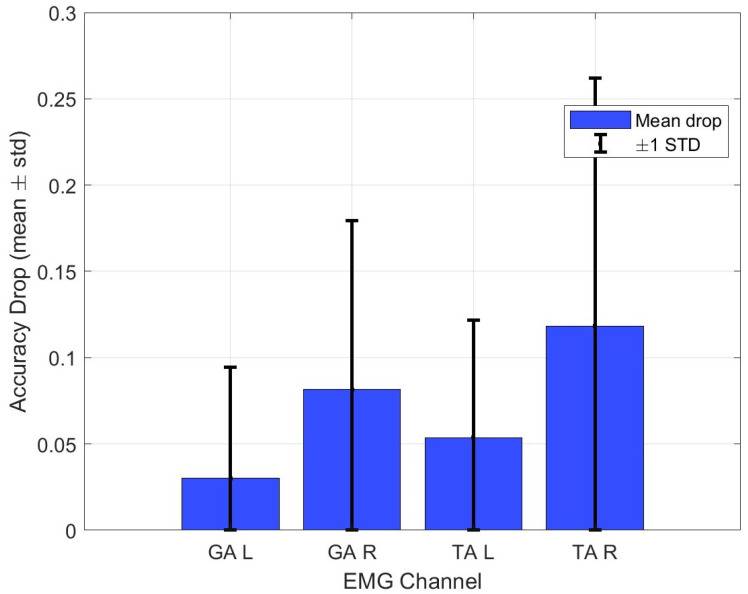
Occlusion sensitivity per muscle channel.

To provide clinicians with a more intuitive sense of which muscle signals drive our model’s decisions, we perform an occlusion sensitivity analysis on each of the four EMG channels. In Figure 8, we plot, for each channel, the mean (±SD) drop in diagnosis accuracy when that channel is set to zero at test time. From the bar-and-error bar chart it is clear that the impact of occluding symmetric channels is not identical, as right side signals tend to matter more for our cohort. Furthermore, TA channels exhibit substantially larger effects on diagnostic accuracy than GA channels. This asymmetry and the dominance of TA over GA agree with clinical observations that tibialis anterior activity during stance and swing phases is a key biomarker of Parkinsonian gait [35,36].

**Figure 9:**
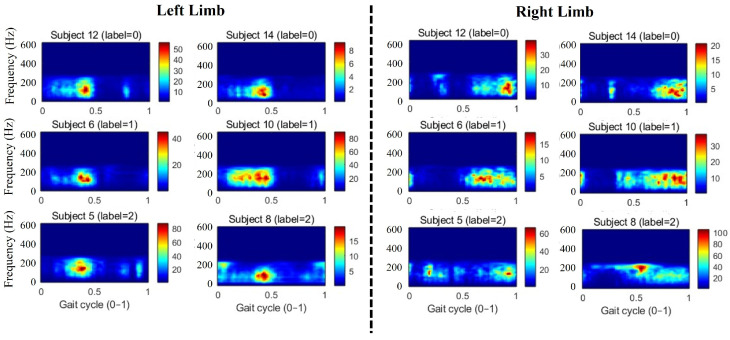
Continuous wavelet coefficient scalograms of the left and right gastrocnemius muscles for subjects 5, 6, 8, 10, 12, and 14.

Figure 9 displays the continuous wavelet coefficient scalograms of the left and right gastrocnemius muscles for the same representative subjects shown in Figure 7. In all participants, the regions of highest wavelet energy are localized to the stance-phase interval and peak at approximately 100 Hz. However, along the time (gait-cycle) axis, healthy controls exhibit tightly concentrated energy bundles, indicating that they require less overall muscle activation to propel the body during stance. In contrast, Parkinson’s patients display a more temporally dispersed distribution of high-energy coefficients—sometimes spanning the entire support phase—suggesting that they must sustain prolonged muscle stimulation to accomplish the same walking task. We propose that these differences in muscular efficiency profiles provide a potent biomarker for the evaluation head.

**Figure 10:**
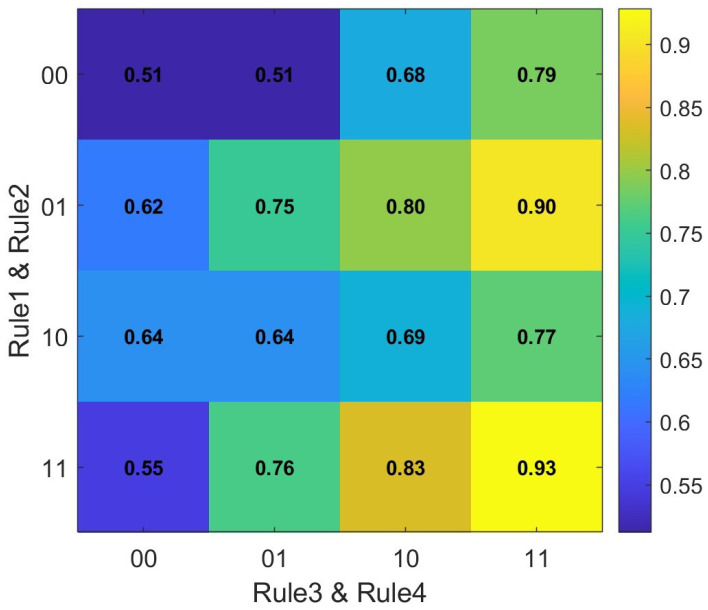
Heatmap of UPDRS level recognition accuracy across all 16 combinations of the four fusion mechanism rules.

To quantify the individual and joint contributions of our four heuristic rules to the final UPDRS level classification, we conduct an ablation study comparing all possible on/off combinations of these rules Figure 10. In total, 16 configurations are evaluated, and their overall recognition accuracies are summarized in the matrix and visualized in the heatmap above. Each row corresponds to the binary state of Rule 1 and Rule 2 (00, 01, 10, 11), and each column corresponds to the binary state of Rule 3 and Rule 4 (00, 01, 10, 11). From the heatmap, it is immediately apparent that disabling all rules (0000) yields the lowest accuracy (0.5119), whereas enabling all four rules (1111) achieves the highest accuracy (0.9286). Moreover, configurations in which at least three rules are active generally outperform those with fewer rules, demonstrating synergistic effects among the heuristics. In particular, the second-row, fourth-column cell (0100 → Rule 3 only disabled) reaches 0.9048, indicating that Rules 1, 2, and 4 together contribute substantially to performance. These results confirm that each rule provides a non-negligible improvement, and that their combined application yields the best UPDRS level classification accuracy.

**Figure 11:**
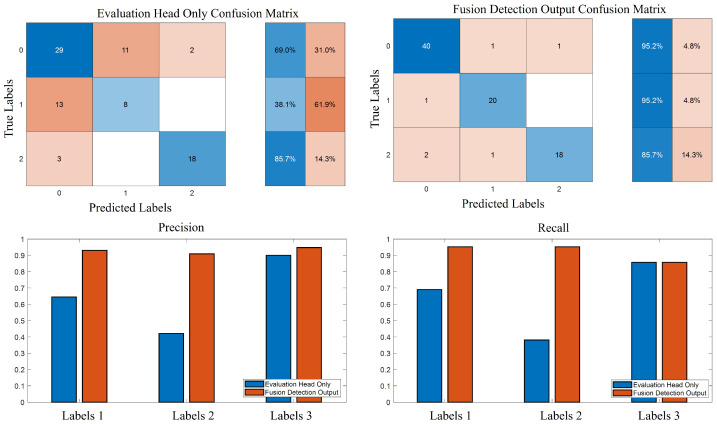
Comparison the outputs of the evaluation head alone with those of the fusion detection framework.

Figure 11 demonstrates the outputs of the evaluation head alone with those of the fusion detection framework. The confusion matrices show that, after fusion detection, the algorithm achieves superior performance both in the binary classification of Parkinson’s disease and in the multi-class UPDRS gait-score prediction. In Table 3, the precision and recall metrics for the fusion detection output exceed those of the evaluation head by itself, confirming that our multimodal integration strategy meaningfully enhances overall scoring accuracy. To demonstrate the statistical significance of fusion over the evaluation head alone, we conducted a paired *t*-test on the 21 fold accuracies, yielding t(20)=6.040, p<0.0001 (mean difference = 0.274, 95% CI [0.179, 0.368]), and a McNemar test on the pooled 84 predictions—where the a=1 sample was corrected by the baseline but misclassified by fusion, and b=24 samples were corrected by fusion but missed by the baseline—giving $\chi^{2}=19.360$, p<0.0001. Both tests confirm the fusion model’s significant performance gain over the single-head baseline.

## 4. Discussion

We validate our bimodal UPDRS gait-scoring framework on 21 participants. In preliminary experiments, the EMG-only wavelet feature head yielded only 69.0% accuracy in identifying healthy controls and 61.9% for Parkinson’s patients. Most misclassifications involved mild cases, including the following: healthy participants wrongly labeled as PD predominantly received score 1, and PD patients misclassified as healthy also tended to be UPDRS 1—indicating errors cluster around mild impairment. Patients at UPDRS-2, with more pronounced gait balance deficits, remains well distinguished from controls.

Incorporating two additional branches (diagnosis head and evaluation head) alongside the balance head, and fusing their outputs, increased classification accuracy for healthy and PD subjects to 95.2 % and 90.5 %, respectively. These results confirm that IMU-derived motion features substantially enhance UPDRS-based scoring precision. Moreover, although the balance head does not increase recall for UPDRS-2 cases, it dose improve precision, reflecting a stricter assessment of gait balance impairment and a reduction in false positives.

Although the UPDRS score itself provides only coarse categorization and may lack sensitivity to very small treatment-induced changes, we observe that our extracted gait kinematic parameters exhibit large effect sizes (Cohen’s d > 0.8) and high statistical power (>0.9) between adjacent UPDRS levels. These robust inter-group differences demonstrate that individual gait metrics—such as stride length, swing/stance ratio, and joint angular excursions—can serve as more sensitive biomarkers for monitoring subtle improvements or early disease progression. Some studies have similarly shown that quantitative gait parameters outperform ordinal UPDRS subitems in detecting incremental motor changes [37].

Table 4 highlights that our multimodal fusion approach achieves a UPDRS gait-scoring accuracy of 92.8 %, substantially outperforming existing single-modality methods. For example, Han et al.’s 2 IMU system reaches only 84.9 % accuracy, and Federico et al.’s 3 IMU-SVM approach yields 66.5 %. Even methods that incorporate complementary modalities—such as Ji et al.’s IMU + pressure-insole setup—achieve 95.4 % only under very controlled conditions and at the cost of additional sensor bulk. In contrast, our lightweight fusion of 2 shank-mounted IMUs and four EMG channels combines the strengths of kinematic and muscle activation signals to deliver both high accuracy and practical wearability. Most prior work remains limited to a single sensing modality (IMU-only, EMG-only, or vision-based), which inherently constrains the discriminative power for nuanced UPDRS grading. By integrating multiple complementary data streams in a unified learning framework, our method not only sets a new accuracy benchmark but also points the way toward everyday, at-home monitoring solutions in which minimally intrusive, multimodal wearables can provide robust, objective UPDRS scoring outside the clinic.

This study has several limitations. First, although our protocol requires only a brief walking task, with considerably less time and effort than traditional UPDRS assessments, it nonetheless imposes new workflow demands as follows: clinicians must be trained in correct sensor placement, calibration procedures, and pre-session setup to ensure reliable data capture. Second, the intermediate outputs (e.g., gait kinematic parameters, wavelet coefficient maps, and EMG envelope traces) are familiar to engineers but may not be immediately interpretable by clinicians without additional training in signal processing and biomechanics, complicating integration with standard tremor and bradykinesia scales. Third, we have only evaluated UPDRS levels 0–2; we did not recruit subjects with level 3 or higher, primarily for safety reasons, and thus, we cannot claim applicability to moderate or severe gait impairment. Future work should extend to advanced patients, perhaps focusing on balance metrics derived from shank IMUs, as these individuals exhibit profound postural instability and often cannot ambulate unassisted. Finally, while we control walking surface and applied static–dynamic calibration to correct mounting errors, we do not systematically assess confounders such as footwear variation, indoor lighting effects on patient comfort or vision, sensor drift over extended use, or EMG electrode misplacement. In clinical settings, where attire is standardized, footwear and lighting may be less critical, but long-term drift could be mitigated by periodic zero-velocity updates at the ankle, and future studies will also explore automated EMG repositioning checks to enhance robustness.

## 5. Conclusions

In summary, we introduce a multimodal EMG–IMU approach for automated UPDRS gait scoring. Our network integrates three specialized branches—a diagnosis head, an evaluation head, and a balance head—followed by a fusion detection module designed to emulate the multi-dimensional assessment process used by clinicians. Validated on 21 participants, the method achieves precisions of 93.0%, 90.9%, and 94.7% for UPDRS levels 0, 1, and 2, respectively. The proposed pipeline is straightforward to implement, significantly reduces the time and labor required for clinical scale assessments, and holds substantial promise for real-world deployment.

## Figures and Tables

**Table 1:** Information of all subjects.

Subjects	UPDRS	Height/cm	Weight/kg	Age	Number
HC	0	170 ± 7	68 ± 8	25 ± 3	10
PD	1	162 ± 5	65 ± 10	62 ± 4	5
PD	2	163 ± 4	63 ± 5	68 ± 7	6

HC denotes Healthy Control, and PD stands for Parkinson’s Disease.

**Table 2:** Cohen’s d and statistical power of gait parameters across UPDRS severity comparisons.

Feature	HC vs. PD1		HC vs. PD2		PD1 vs. PD2	
	* d *	Power	* d *	Power	* d *	Power
SL/H	1.34	0.996	2.46	1.000	1.08	0.707
GC	0.26	0.176	–0.34	0.656	–0.39	0.235
SPR	0.23	0.095	1.46	0.996	1.45	0.999
MGS	0.73	0.503	–0.13	0.403	–0.23	0.966
MFC/H	0.56	0.300	0.47	0.334	0.03	0.054
SR (rad)	1.63	1.000	2.74	1.000	1.02	0.591

**Table 3:** Cross-validation performance (21 folds) of baseline (evaluation-head only) vs. fusion model.

Metric	Baseline (Mean ± Std, 95% CI)	Fusion (Mean ± Std, 95% CI)
Overall Accuracy	0.655 ± 0.185, [0.571, 0.739]	0.929 ± 0.140, [0.865, 0.992]
Class 0 Precision	0.667 ± 0.255	0.952 ± 0.120
Class 0 Recall	0.690 ± 0.370	0.952 ± 0.150
Class 1 Precision	0.417 ± 0.359	0.929 ± 0.239
Class 1 Recall	0.381 ± 0.498	0.952 ± 0.218
Class 2 Precision	0.944 ± 0.162	0.972 ± 0.118
Class 2 Recall	0.857 ± 0.359	0.857 ± 0.359

**Table 4:** Results from related work.

Researcher	Performance	Hardware Platform	Subjects	Methods
Urs [20]	R = 0.739	8 EMG	45	Random Forest
Han [22]	84.9%	2 IMU	53	Nonlinear Models
Federico [38]	66.5%	3 IMU	34	Support Vector Machine
Endo [39]	79%	Camera	54	Transformer Models
Ji [40]	95.35%	1 IMU + 1 pressure sensor	50	Support Vector Machine
This paper	92.8%	2 IMU + 4 EMG	21	Multi-head Fusion Classification

## Data Availability

The data presented in this study are available on request from the corresponding author due to the ongoing collaborative nature of the project with the hospital.
